# Extensive historical and contemporary hybridization suggests premating isolation in *Vermivora* warblers is not strong: A reply to Confer et al.

**DOI:** 10.1002/ece3.7327

**Published:** 2021-06-17

**Authors:** David P. L. Toews, Marcella D. Baiz, Gunnar R. Kramer, Irby J. Lovette, Henry M. Streby, Scott A. Taylor

**Affiliations:** ^1^ Department of Biology Pennsylvania State University University Park PA USA; ^2^ Department of Environmental Sciences University of Toledo Toledo OH USA; ^3^ Fuller Evolutionary Biology Program Cornell Lab of Ornithology Cornell University Ithaca NY USA; ^4^ Department of Ecology and Evolutionary Biology University of Colorado Boulder Boulder CO USA

## Abstract

We present comments on an article published by Confer et al. (Ecology and Evolution, 10, 2020). Confer et al. (2020) aggregate data from multiple studies of social pairing between *Vermivora chrysoptera* and *V. cyanoptera*, two wood warblers in the family Parulidae that hybridize extensively where they co‐occur. From analysis of these data, they conclude there is near‐complete reproductive isolation between these two species. In our reply, we show that this finding is not supported by other lines of evidence, and significant drawbacks of their study design preclude such strong conclusions. In our critique, we show that (a) coarse‐scale plumage classifications cannot be used to accurately estimate hybrid ancestry in *Vermivora*; (b) extra‐pair paternity is very high in *Vermivora* and is likely facilitating hybridization, yet was not considered by Confer et al. (2020), and we suggest this will have a substantial influence on the interpretation of reproductive isolation in the system; and (c) the central finding of strong total reproductive isolation is not compatible with the results of other long‐term studies, which demonstrate low isolation and high gene flow. We conclude with a more comprehensive interpretation of hybridization and reproductive isolation in *Vermivora* warblers.

## Graphical abstract

*Vermivora* warblers—golden‐winged and blue‐winged warblers—hybridize extensively where they co‐occur. We discuss concerns regarding recent suggestions that this extensive hybridization takes place in the face of near complete pre‐mating isolation, which we suggest is not consistent with other data.

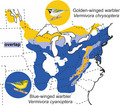

## 1 DISCUSSION

**FIGURE 1:**
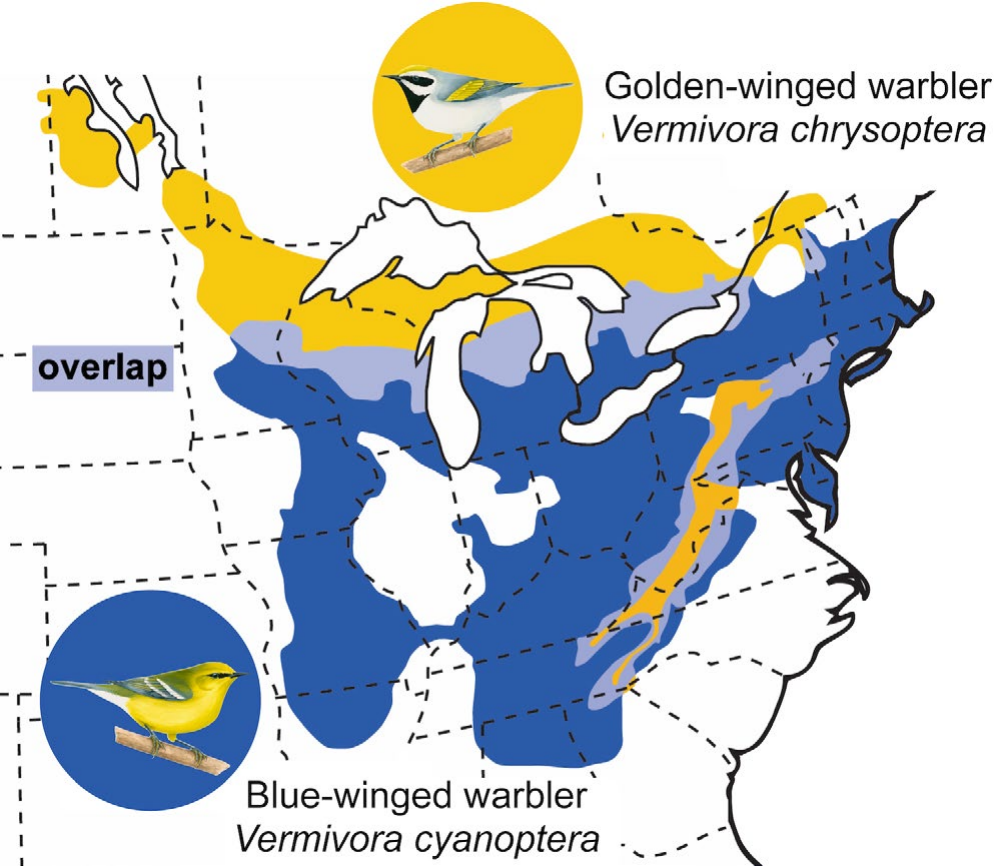
The range of golden‐winged (orange) and blue‐winged (blue) warblers. Areas of overlap (light blue) have both of the parental phenotypes, as well as birds with hybrid phenotypes. Illustrations of the parental phenotypes by Liz Clayton Fuller

Understanding the barriers to reproduction in closely related species is a central goal of evolutionary biology. Hybrid zones are particularly important as, by definition, they occur only between taxa that are not completely reproductively isolated. Thus, they allow for an investigation of the specific traits, genes, and ecological and behavioral settings that may contribute to partial isolation among groups. Among avian hybrid zones, one of the more perplexing cases of extensive hybridization occurs between two wood warblers (Parulidae), golden‐winged and blue‐winged warblers (*Vermivora chrysoptera* and *V. cyanoptera,* respectively*)*. These two species form a mosaic hybrid zone (Figure 1), and, while they are phenotypically divergent in their plumage, they are nearly indistinguishable at the genomic level (Gill, 1997; Toews et al., 2016; Vallender et al. 2007). Hybridization between them has been documented for over a century and is so extensive that it has led to conservation concern for the persistence of the less abundant and declining *V. chrysoptera*, although the mechanistic causes of these declines are likely multi‐causal (e.g., Kramer et al. 2018).

Fieldwork on these birds is challenging, but a number of research teams have monitored pairs and examined nesting success under natural conditions. Confer et al. (2020) aggregate these field data from multiple studies to present an analysis of social pairing patterns in *Vermivora,* with the stated goal of providing greater insights into the extent of reproductive isolation in areas of breeding sympatry. The authors use long‐term observational data based on plumage classifications of social pairs following traditional plumage scoring methods. Unfortunately, we now know through extensive genomic studies, beginning in 2016, that these coarse‐scale plumage classifications are not indicative of hybrid status (Baiz et al. 2020; Toews et al. 2016). There are also important statistical baises introduced by pooling data derived from different populations and time periods, as reviewed by Moura et al. (2021). They then use these pairing metrics to estimate the extent of behavioral isolation and sexual selection against hybrids. Unfortunately, their central finding of “near‐complete levels of reproductive isolation” between species is not supported by other lines of evidence, and significant drawbacks of their own study design preclude their strong conclusions.

Here we address the limitations of their approach. We structure our critique in three parts and conclude with a more nuanced and comprehensive interpretation of hybridization and reproductive isolation in *Vermivora* warblers.

### 1.1 Plumage cannot be used to estimate hybrid ancestry in *Vermivora*

Confer et al. (2020) focus on “primary hybridization,” which they describe as the mating of “genetically pure” *V. chrysoptera* and *V. cyanoptera*. In this system, several classes of hybrids have plumage characteristics distinct from both parental types and have traditionally been named (e.g., “Brewster's warbler” is a hybrid phenotype long associated with typical first‐generation [F_1_] hybrids). Yet, the idea of “genetic purity” is problematic in *Vermivora*, because genomic data show clearly that historical gene flow between these parental species has been extensive for hundreds—possibly thousands—of generations, resulting in the homogenization of nearly all differences in their genomes (Toews et al. 2016). Moreover, as first documented by Faxon (1913), the hybrids *themselves* can produce offspring with “pure” phenotypes: “The young birds of mixed parentage were absolutely pure in plumage—either Brewster's warblers or *Golden‐wings*.” Thus, what does “genetic purity” mean in this context, if hybrids themselves can recapitulate the “pure” parental forms?

Recent genetic data from *Vermivora* also suggest hybrid ancestry cannot be accurately predicted from plumage traits alone, and several of these traits change with age (Baiz et al. 2020). Ancestry analysis from Baiz et al. (2020) found that none of the six “Brewster's” warblers analyzed were F_1_ hybrids, refuting the assumptions of Confer et al. (2020) that “Brewster's” warblers are the direct products of “primary hybridization.” Moreover, all of the “pure” parental phenotypes sequenced by Toews et al. (2016) showed extensive genomic homogeneity, suggesting high rates of historical introgression and little evidence that genomically “pure” *V. chrysoptera* and *V. cyanoptera* actually exist. Therefore, without genotypes of social pairs, reliable inferences cannot be drawn about rates of introgression and reproductive isolation from plumage classifications, as *all* “pure” birds also likely have significantly admixed ancestry. Unfortunately, this issue of cryptic admixture is perhaps particularly acute for birds in areas of sympatry where both species currently breed and hybridize, meaning that the field settings where phenotypic pairing ratios can be calculated are also those most likely to be confounded by this underlying unreliability of using plumage traits to infer ancestry.

### 1.2 Extra‐pair paternity is very high in Vermivora, likely facilitating hybridization

Extra‐pair copulations (EPCs) have been well documented for both species and have been suggested as an important context to understand hybridization in *Vermivora* (Hartman et al. 2012). Vallender et al. (2007)—who studied a population comprised of primarily phenotypic *V. chysoptera* and intermediate hybrid phenotypes—found that >30% of nestlings and >55% of broods were results of extra‐pair matings. Quantifying the role that EPCs play in contributing to hybridization is difficult, and Confer et al. (2020) acknowledge this limitation in their text. However, they suggest that “social pairing data should only produce biased estimates of behavioral isolation if individuals systematically seek extra‐pair partners that differ in phenotype from their social partner.” This is an incorrect assertion. Consider, for example, a scenario where EPCs are random with respect to phenotype; this null scenario will significantly reduce assortative mating. In other words, strict dis‐assortative mating is not required to promote hybridization. Confer et al. (2020) suggest that their analyses would be “minimally confounded” by the presence of EPCs, yet logic would dictate that it would have a substantial influence on the interpretation of total reproductive isolation given the many opportunities for EPCs at their study sites. Furthermore, in other hybridizing birds where EPCs have been tracked under natural conditions, females of both forms prefer extra‐pair mates of one species' plumage phenotype (e.g., Baldassarre & Webster, 2013).

### 1.3 The findings of strong total reproductive isolation are not compatible with long‐term studies

The central claim from Confer et al. (2020) is that reproductive isolation is “near‐complete” (i.e., 0.96) between *V. chrysoptera* and *V. cyanoptera*. It is then fair to ask how does this compare with dynamics observed in other populations in the hybrid zone that have been studied over the long term? Bennett et al. (2017) documented detailed phenotypic change in a single, well‐mixed population of *Vermivora* from 2008–2015 at Fort Drum, New York. At the beginning of the study, the phenotypes were approximately 50% *V. chrysoptera*, 35% *V. cyanoptera*, and 15% phenotypic hybrids. By the end of the study less than a decade later, the proportions had changed significantly: 30% *V. chrysoptera*, 50% *V. cyanoptera*, and 10%–20% hybrids, with consistent directional changes in each study year. This is a pattern that has played out, similarly asymmetrically, across many populations over at least the past half‐century, as breeding *V. cyanoptera* have consistently replaced disappearing *V. chrysoptera* across much of northeastern North America, with an intermediate stage involving substantial hybridization (Gill, 1980).

These dynamics are not consistent with a scenario of high reproductive isolation between these forms, which would instead predict low or inconsistent hybrid zone movement, phenotypic stability, and low levels of gene flow. Indeed, the vast majority of the reproductive isolation in Confer et al. (2020) is attributed to premating isolation, in the form of assortative social pairing. However, through simulations, both Pulido‐Santacruz et al. (2018) and Irwin (2020) find that even *if* assortative mating in sympatry is high, premating isolation in hybrid zones is surprisingly ineffective at maintaining isolation.

Confer et al. (2020) do note an intriguing reduction in pairing frequencies of one of the named hybrid phenotypes (“Brewster's warblers”), possibly consistent with reduced phenotypic hybrid fitness (although it will be important to understand how this relates to reproductive success of hybrids when EPCs are eventually considered). While the magnitude of any fitness reduction in “Brewster's” is not large, as Irwin (2020) details in the context of a “tension zone,” a large reduction in hybrid fitness is not necessary for a hybrid zone to remain stable. As Bennett et al. (2017) show, however, the hybrid zone between *V. chrysoptera* and *V. cyanoptera* is not stable—at least in the eastern portion of the *Vermivora* distribution—and thus, even this small reduction in hybrid pairing cannot maintain the species differences. We therefore posit that extensive mixing in areas of sympatry is more consistent with low levels of total reproductive isolation—that is, both low pre‐ and postmating isolation—and results in high gene flow. Low levels of reproductive isolation are further supported by multiple lines of evidence, including genomic analyses where there is evidence of extensive historical introgression (Toews et al. 2016).

### 1.4 We still have much to learn about isolation in *Vermivora*

As researchers who have studied *Vermivora* extensively, we appreciate that Confer et al. (2020) sought to address a key paradox of this system: How could such distinct phenotypes be maintained in the face of high gene flow? Confer et al. (2020) imply this distinctness is largely a result of strong behavioral premating reproductive isolation; we have outlined here a subset of the issues involved in arriving at this interpretation based on social pairing data from plumage phenotypes alone.

We suggest alternative considerations. The first possibility is that the parental phenotypes are not actually being “maintained,” or will not for much longer, given the drastic declines in some populations of *V. chrysoptera* over the past half‐century. The main driver of this decline is likely wintering habitat loss (Kramer et al. 2018). Asymmetric “genomic extinction” from hybridization is probably contributing, although asymmetric introgression could also result from a directional change in relative abundance even with random or symmetric hybridization. Analyses have suggested that hybridization has been ongoing for many hundreds of generations within *Vermivora* (Toews et al. 2016); thus, the suggestion by Confer et al. (2020) that conservation actions could somehow “repair” isolation between the species is both misplaced and unfeasible.

Confer et al. (2020) imply that part of their motivation to emphasize isolation between these species is a concern over their taxonomic treatment, noting that any “decision regarding listing [under the Endangered Species Act] will be highly influenced” by their taxonomic status (i.e., recognized as a single species vs. two species). However, valid concerns about potential protection status should not motivate the production of research that contradicts the preponderance of available evidence, nor should conservation considerations drive taxonomic classification. Moreover, such concerns are not rooted in Endangered Species Act implementation. The “regulatory frameworks [in both the USA and Canada] support the conservation of evolutionary significant variation within species” (Toews et al. 2016), and populations of conservation concern of both *V. chrysoptera* and *V. cyanoptera* exhibit phenotypic variation that could qualify as evolutionarily significant units under law. Additionally, regardless of the species status of *V. chrysoptera*, range‐wide endangerment status is unlikely because its breeding population stronghold in northern Minnesota, and increasingly in southern Manitoba, has experienced long‐term positive population growth, even as most more easterly populations have declined or vanished.

Finally, while we emphasize that we still have much to learn about hybridization dynamics in *Vermivora* warblers, the most balanced interpretation of the extensive available data suggests that premating isolation is not sufficient to maintain differences between these species. That said, the few restricted genomic regions that are fixed between *V. chrysoptera* and *V. cyanoptera* might be involved in some currently undescribed reproductive barriers. Dramatic plumage polymorphisms have been maintained in other avian systems, implicating balancing selection in some instances (e.g., face‐color polymorphisms in Gouldian finches, Kim et al. 2019) and in some cases this has been facilitated by atypical mating systems and chromosomal rearrangements (e.g., the divergent male phenotypes in Ruffs; Lank et al. 1995). It is possible that analogous processes are playing out in *Vermivora* warblers. We suggest that careful field and genetic study of pedigreed individuals within the hybrid zone over multiple generations has the greatest potential to advance our understanding of reproductive isolation and its implications in this fascinating and charismatic system.

## CONFLICT OF INTEREST

The authors have no conflicts of interests to declare.

## AUTHOR CONTRIBUTIONS

**David P. L. Toews:** Conceptualization (equal); Writing‐original draft (lead); Writing‐review & editing (equal). **Marcella D. Baiz:** Conceptualization (equal); Writing‐original draft (equal); Writing‐review & editing (equal). **Gunnar R. Kramer:** Conceptualization (equal); Writing‐original draft (equal); Writing‐review & editing (equal). **Irby J. Lovette:** Conceptualization (equal); Writing‐original draft (equal); Writing‐review & editing (equal). **Henry M. Streby:** Conceptualization (equal); Writing‐original draft (equal); Writing‐review & editing (equal). **Scott A. Taylor:** Conceptualization (equal); Writing‐original draft (equal); Writing‐review & editing (equal).

## ETHICAL APPROVAL

All work by the authors has been previously approved by their respective institutional animal care committees.

## Data Availability

No data were directly generated in the production of this letter.
